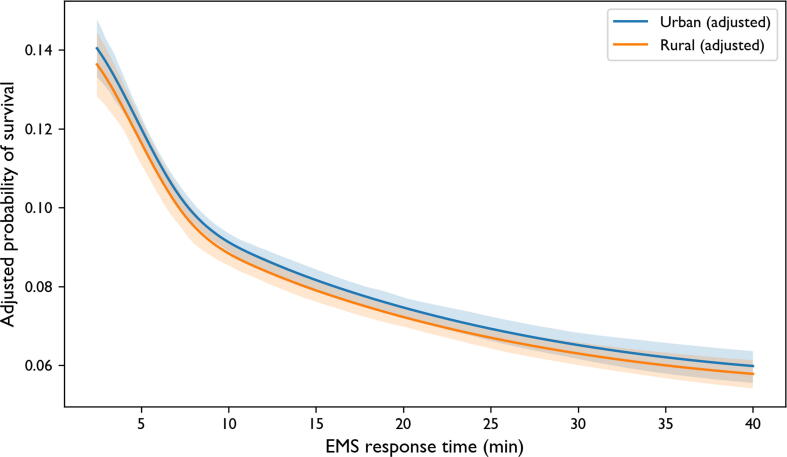# Urban-rural disparities in out-of-hospital cardiac arrest outcomes: a nationwide Hungarian study

**DOI:** 10.1016/j.resplu.2025.101108

**Published:** 2025-09-23

**Authors:** Ádám Pál-Jakab, Bettina Nagy, Boldizsár Kiss, György Pápai, Nora Boussoussou, Béla Merkely, Miklós Constantinovits, Gábor Csató, Péter Sótonyi, Brigitta Szilágyi, Endre Zima

**Affiliations:** a Heart and Vascular Centre, Semmelweis University, 1122 Budapest, Hungary; b Hungarian National Ambulance Service (HNAS), Markó Street 22., 1055 Budapest, Hungary; c Budapest University of Technology and Economics, Műegyetem rkp. 3, 1111 Budapest, Hungary; d Corvinus University of Budapest, Fővám tér 8, 1093 Budapest, Hungary; e Anaesthesiology and Perioperative Care Institute, Semmelweis University, Hungary

**Keywords:** Cardiac arrest (CA), Cardiopulmonary resuscitation (CPR), Urban-rural disparity, Emergency medical services (EMS), Out-of-hospital cardiac arrest (OHCA)

## Abstract

**Background:**

Out-of-hospital cardiac arrest (OHCA) outcomes often differ between urban and rural settings, but comprehensive nationwide data from Central-Eastern Europe using uniform data collection and modern confounding control remain limited. We investigated urban–rural disparities in OHCA outcomes in Hungary.

**Methods:**

We analysed 130,258 OHCA cases (2018–2023) from the Hungarian National Ambulance Service registry, classified as urban (70.1 %) or rural (29.9 %) using national administrative categories. The primary outcome was on-scene return of spontaneous circulation (ROSC). We performed univariable and multivariable logistic regression, propensity score matching (PSM) and continuous response-time modeling using natural cubic splines.

**Results:**

The overall ROSC rate was 9.1 % (urban: 9.4 %, rural: 8.3 %, p < 0.001). After PSM, urban location remained significantly associated with higher survival (OR = 1.26, 95 % CI 1.20–1.32, p < 0.001). EMS response times were significantly longer in rural areas (median 14.9 vs 9.8 min, p < 0.001). Urban survival advantage was most pronounced in cases with shockable rhythms (OR = 1.57, 95 % CI 1.43–1.72), medical-witnessed arrests (OR = 1.31, 95 % CI 1.20–1.42), and response times ≤8 min (OR = 1.59, 95 % CI 1.44–1.76).

**Conclusions:**

Significant urban–rural disparities in OHCA on-scene ROSC persist even after accounting for patient and arrest characteristics. These findings highlight the need for targeted interventions to strengthen the Chain of Survival in rural communities.

## Introduction

Out-of-hospital cardiac arrest (OHCA) remains a significant public health challenge, with high mortality rates and variable outcomes influenced by regional healthcare disparities. Across Europe, OHCA incidence ranges from 67 to 170 cases per 100,000 people, reflecting differences in emergency medical service (EMS) capabilities, healthcare organisations, and community health profiles.^1^, ^2^, ^3^, ^4^, ^5^, ^6^ In particular, survival decreases by approximately 10 % with each minute delay in cardiopulmonary resuscitation (CPR) when the initial rhythm is shockable, underscoring the critical importance of an efficient and fast EMS response.^7^, ^8^ Non-shockable rhythm cases have a much lower survival rate.

Despite international efforts to document urban–rural differences in OHCA outcomes,^9^ nationwide analyses from Central–Eastern Europe using uniform data collection and modern confounding control remain limited.^3^, ^10^, ^11^ To address this gap, we analyzed the Hungarian National Ambulance Service registry, which captures all EMS-attended OHCAs nationwide from 2018 to 2023. Our study combines (i) propensity score matching to reduce confounding, (ii) multivariable logistic regression to quantify adjusted associations, and (iii) continuous modeling of EMS response time using natural cubic splines to avoid information loss from coarse categorization. These methodological advances allow us to more accurately isolate the effect of location on OHCA outcomes and provide actionable evidence for targeted EMS interventions.

Hungary's geographic and healthcare context provides an informative setting for examining urban–rural OHCA disparities. The country has a total area of 93,030 km^2^ with heterogenous settlement patterns and population density, yet OHCA care is delivered by a single, unified national EMS provider with standardized protocols, equipment, and training across regions.^12^, ^13^ This geographic diversity, combined with population density variations ranging from 3350 inhabitants/km^2^ in Budapest to <50 inhabitants/km^2^ in rural counties, creates natural variations in EMS accessibility while maintaining system-level standardization—providing an ideal natural experiment for isolating geographic effects on OHCA outcomes.^14^

## Materials and methods

### Aims of the study

This study aimed to investigate the urban–rural disparities in OHCA outcomes through the first comprehensive nationwide analysis of all OHCA cases in Hungary. We proposed to identify the key factors contributing to on-scene survival rates in urban and rural settings while quantifying the independent effect of location after adjusting for patient demographics, arrest characteristics, and emergency medical service response variables.

### Study design and setting

This population-based, retrospective observational study analysed all OHCA cases in Hungary from January 2018 to December 2023 (Supplementary Table 1). Raw data were obtained from the mandatory reports of the Hungarian National Ambulance Service (HNAS), the only EMS provider in Hungary, handling approximately 1.3 million emergency calls annually. The study was conducted and reported according to the STROBE^15^ guidelines for observational studies.

This study was approved by the Hungarian Medical Research Council (IV/3043/2021/EKU and IV/3043-3/2021/EKU), adhered to GDPR compliance regulations, and was conducted in accordance with the Declaration of Helsinki.

### Consent

Due to the retrospective nature of using anonymised registry data, individual informed consent was waived by the ethics committee, in accordance with national regulations for registry-based research.

### Patient population and selection

All adult OHCA cases (≥18 years) attended by EMS during the study period were eligible for inclusion. We excluded cases related to self-harm or trauma, and cases where survival outcome was not recorded. Cases with EMS response times exceeding 120 min were also excluded as statistical outliers, representing <0.01 % of the total dataset. After applying these criteria, the final analytical cohort consisted of 130,258 OHCA cases, representing 99.4 % of the original dataset available for analysis.

OHCA locations were classified as urban or rural based on the country’s official administrative settlement categories,^12^ which integrate legal status, historical development, and functional roles. Urban areas encompassed the capital city (Budapest), cities with county rights, and other towns granted city status, while rural areas included villages and large villages. This classification corresponds to substantial population density differences: Budapest (3350 inhabitants/km^2^), other urban areas (average 450 inhabitants/km^2^), and rural villages (average 45 inhabitants/km^2^), creating natural variations in healthcare accessibility and emergency response infrastructure.^14^ Based on Hungary’s Central Statistical Office (KSH) data for January 1, 2023, Budapest had a population of 1,671,004; other cities had 5,067,717 inhabitants; and villages accounted for 2,861,023 residents out of a total national population of 9,599,744.^12^

### Data collection

Data were extracted from randomly anonymized HNAS electronic health records. Variables included demographics (age, sex), clinical factors (initial rhythm, etiology), circumstantial information (location, bystander witness status), interventions (bystander CPR, AED use, advanced airway management, mechanical chest compression device usage), call daytime interval, and EMS total response time. The primary outcome was the return of spontaneous circulation (ROSC) on-scene.

### Emergency medical service characteristics

The HNAS operates as a unified, nationwide system with standardized protocols across all regions. All ambulance crews have advanced life support accreditation and are trained according to national guidelines, in conformity with international standards (ERC and ILCOR), with scope including advanced airway management, intravenous/intraosseous medication administration, and manual/automated defibrillation. Equipment specifications and training requirements are set centrally and applied in both urban and rural areas, minimizing crew-level variation that might confound urban–rural comparisons.

### Statistical analysis

Analyses were conducted by using Visual Studio Code (version 1.99.0–insider [Universal]) and Python (version 3.8).^16^, ^17^ Descriptive statistics were calculated separately for the entire cohort and for the urban and rural subgroups. Mann-Whitney *U* test for continuous variables and Chi-square test for categorical variables were used for between-group comparisons. Missing data patterns were assessed for all variables, and cases with missing values for key outcome or exposure variables were excluded from the analysis using complete case analysis. The proportion of missing data was minimal, with fewer than 2 % of cases having missing values for any single variable. Continuous variables were assessed for normality using visual inspection of histograms and Q-Q plots. Data that were normally distributed (e.g., age) were presented as mean ± standard deviation, while skewed data (e.g., EMS response times) were presented as median and interquartile range (IQR).

EMS response times were analyzed both categorically and continuously to address different analytical objectives. Categorical analysis (≤8, 8–15, >15 min) used clinically relevant thresholds based on established evidence that response within 8 min optimizes survival while delays beyond 15 min markedly reduce survival rates.^18^, ^19^, ^20^ However, to address potential information loss from categorization and detect non-linear patterns, we conducted continuous modeling using natural cubic splines (3 degrees of freedom) with rural × spline interaction terms. This approach preserves all temporal information while allowing detection of time-dependent urban–rural effects across the entire response spectrum.

Emergency calls were categorised by the time of day into three 8-hour periods: morning (0–8), daytime (8–16), and evening (16–24).

Univariable and multivariable logistic regression were developed using variables with established clinical relevance from prior literature.^2^, ^21^ These variables included age, sex, location, arrest etiology, initial cardiac rhythm, bystander witness status, EMS response time, arrest location, bystander interventions (CPR, AED use), and EMS interventions (advanced airway, mechanical chest compression). From these clinically relevant variables, high-priority variables for exact matching were selected based on strongest confounding potential and clinical importance: bystander-witnessed status, arrest etiology, while age matching was performed by exact matching on individual years of age. Initial cardiac rhythm, while clinically crucial for outcomes, was not exact-matched to preserve sufficient sample size for rhythm-specific subgroup analyses. Variance inflation factors were calculated to assess multicollinearity.^22^

To minimise confounding factors, propensity score matching was performed using a 1:1 nearest-neighbour approach with a caliper width of 0.2 standard deviations. Standardised mean differences were calculated to assess covariate balance before and after matching.

Cardiac etiology was determined based on clinical presentation, circumstances of arrest, and available medical history as recorded by attending EMS personnel following standardized protocols. Cases witnessed by medically trained persons were defined as on-duty EMS personnel, physicians, nurses, or other certified healthcare professionals. 'Unknown etiology' specifically refers to cases where insufficient information prevented etiology determination despite systematic clinical assessment. While recent Utstein guidelines^23^ recommend coding unknown cases as 'presumed cardiac,' our registry maintains this distinction for three reasons: (1) to preserve clinical granularity for quality improvement initiatives, (2) to avoid potential misclassification bias in epidemiological analyses where etiology serves as an important confounder, and (3) to enable comparison with earlier registry periods using consistent definitions.

Sensitivity analysis modeled EMS response time continuously using natural cubic splines (degrees of freedom = 3) with rural × spline interaction terms to explore effect modification across the response time spectrum, avoiding information loss inherent in categorical approaches.

Model performance was evaluated by using the area under the curve in the receiver operating characteristic (AUC) with 95 % confidence intervals. Internal validation was conducted by using a 10-fold cross-validation with 5 repetitions and bootstrap validation (1000 iterations) to correct for prediction overfitting. Model calibration was assessed by using calibration plots and Hosmer-Lemeshow goodness-of-fit tests.^24^

Subgroup analyses were performed based on initial cardiac rhythm, bystander-witnessed status, and age groups. Sensitivity analyses explored the effects of varying EMS response time thresholds and different propensity score matching parameters. Focused response time threshold analysis was conducted to identify critical time points where urban–rural survival disparities were most pronounced. For multiple predefined thresholds (5, 8, 10, 15, 20, and 30 min), cases were categorised as “fast response” (≤threshold) or “slow response” (>threshold), with survival rates and urban–rural odds ratios calculated for each category.^25^, ^26^, ^27^, ^28^

## Results

### Cohort and baseline characteristics

After exclusion criteria were applied, 130,258 OHCA cases were included in the final analysis, 91,341 cases (70.1 %) that occurred in urban areas and 38,917 (29.9 %) in rural settings.

The overall mean age was 69.6 ± 14.2 years (mean ± standard deviation). Rural settings had a higher proportion of patients aged ≤65 years (39.9 % vs 33.9 % in urban areas, p < 0.001) and a greater percentage of males (60.6 % vs 57.8 %, p < 0.001). Cardiac causes were more prevalent in rural cases (26.0 % vs 21.4 % in urban ones, p < 0.001), whereas cases with unknown etiology were more common in urban settings (45.5 % vs 38.5 %, p < 0.001).

Rural areas had higher rates of cases witnessed by medically trained persons (defined as on-duty EMS personnel, physicians, nurses, or other certified healthcare professionals) (18.9 % vs 14.6 % in urban areas, p < 0.001) and non-medical bystander-witnessed events (40.4 % vs 35.4 %, p < 0.001). Initial cardiac rhythm distributions showed higher asystole prevalence in rural settings (71.3 % vs 69.4 % in urban areas, p < 0.001).

### EMS response times and arrest characteristics

EMS response time was significantly longer in rural areas (median 14.9 min [IQR: 11.2–20.1] vs 9.8 min [IQR: 6.5–15.6] in urban settings, p < 0.001), representing a 52.0 % longer median response time. The proportion of cases with response times ≤8 min was substantially higher in urban (37.6 %) versus rural (8.8 %) settings.

Most arrests occurred at home (80.5 %), with a higher proportion in rural areas (81.7 %) compared to urban areas (80.0 %, p < 0.001), whereas public location arrests were more frequent in urban settings (p < 0.001) (Supplementary Table 1).

### Primary outcome: ROSC rates and urban-rural disparities

The overall ROSC rate on-scene was 9.1 % (95 % CI 8.9–9.2 %), with significant urban–rural disparities (9.4 % [95 % CI 9.2–9.6 %] in urban vs 8.3 % [95 % CI 8.1–8.6 %] in rural areas, p < 0.001) (Supplementary Table 2). This disparity persisted across most subgroups. The urban–rural survival gap was most pronounced in younger patients (12.6 % vs 10.6 %, p < 0.001), medical staff-witnessed arrests (19.7 % vs 14.7 %, p < 0.001), and cases with VF/VT as initial rhythm (25.4 % vs 21.6 %, p < 0.001), respectively.

Analysis of EMS response time categories showed that 12.0 % of patients with response times of ≤8 min achieved ROSC, compared to 8.9 % in the 8–15-min category and 6.7 % in the >15-min category. The urban survival advantage was significant for response times of ≤8 min (12.1 % vs 10.8 %, p = 0.029), whereas intermediate response times of 8–15 min showed higher survival rates in rural areas (8.5 % vs 9.8 %, p < 0.001). No significant difference was observed for prolonged response times (>15 min: 6.8 % vs 6.7 %, p = 0.678).

The time of day analysis showed variations in survival rates across EMS call times, with calls during daytime hours (8–16) showing higher survival rates (9.7 %, 95 % CI 9.4–9.9 %) compared to morning (0–8) hours (7.2 %, 95 % CI 7.0–7.5 %). The urban–rural survival gap was consistent across all time periods, with the largest disparity observed during evening hours (16–24) (Supplementary Table 2).

### Analysis of factors associated with survival

In the univariable analysis, rural location was associated with significantly lower odds of survival (OR = 0.88, 95 % CI 0.84–0.92, p < 0.001) (Table 1). Other significant factors associated with survival included initial cardiac rhythm apart from asystole, which showed the strongest association: VF/VT (OR = 6.22, 95 % CI 5.93–6.52), bradycardia (OR = 4.52, 95 % CI 3.92–5.22), and pulseless electrical activity (PEA) (OR = 3.38, 95 % CI 3.22–3.54) all demonstrating significantly higher odds of survival compared to asystole (all p < 0.001). Bystander defibrillation by AED conferred substantial benefit (OR = 10.51, 95 % CI 9.17–12.05, p < 0.001), and medical-witnessed arrests had significantly better outcomes than unwitnessed or bystander-witnessed events. Mechanical chest compression device usage showed a significant positive association with survival in the univariable analysis (OR = 1.64, 95 % CI 1.57–1.70, p < 0.001). Detailed univariable and multivariable regression results for urban and rural populations are provided in Supplementary Tables 3 and 4.

**Table 1:** Overall Cohort – Univariable and Multivariable Logistic Regression Results for Factors Associated with Survival.

**Category**	**Variable**	**Level (Reference)**	**Univariable OR (95 % CI)**	**p-value (Uni)**	**Multivariable OR (95 % CI)**	**p-value (Multi)**
**Patient Variables**	**Age**	*≤65*	*Ref.*	*–*	*–*	*–*
		>65	0.59 (0.57–0.62)	p < 0.001	0.64 (0.61–0.66)	<0.001
	**Etiology**	*Cardiac*	*Ref.*	*–*	*–*	*–*
		Internal Medical Disease	0.65 (0.62–0.68)	<0.001	0.78 (0.74–0.82)	<0.001
		Non Cardiac	0.81 (0.76–0.87)	<0.001	1.02 (0.94–1.10)	0.64
		Unknown	0.13 (0.12–0.14)	<0.001	0.42 (0.39–0.45)	<0.001
	**Sex**	*Male*	*Ref.*	*–*	*–*	*–*
		Female	0.94 (0.90–0.97)	<0.001	1.22 (1.17–1.27)	<0.001

**Location & Time**	**Arrest Location**	*Public*	*Ref.*	*–*	*–*	*–*
		Residence (Home)	0.43 (0.41–0.45)	<0.001	0.55 (0.52–0.57)	<0.001
		Other	0.67 (0.63–0.72)	<0.001	0.68 (0.63–0.74)	<0.001
	**Daytime hours of Arrest**	*0–8*	*Ref.*	*–*	*–*	*–*
		8–16	1.37 (1.31–1.44)	<0.001	1.07 (1.01–1.12)	0.016
		16–24	1.37 (1.30–1.45)	<0.001	1.08 (1.03–1.15)	0.004
	**Urbanisation Level**	*Urban*	*Ref.*	*–*	*–*	*–*
		Rural	0.88 (0.84–0.92)	<0.001	0.83 (0.79–0.87)	<0.001

**Bystander Variables**	**Bystander Witness**	*Medical*	*Ref.*	*–*	*–*	*–*
		Non-Medical	0.59 (0.57–0.62)	<0.001	0.56 (0.53–0.59)	<0.001
		None	0.20 (0.19–0.22)	<0.001	0.46 (0.43–0.49)	<0.001
	**Bystander AED Use**	*No*	*Ref.*	*–*	*–*	*–*
		Yes, Non-Shockable Rhythm	2.32 (2.07–2.60)	<0.001	1.18 (1.04–1.35)	0.01
		Yes, Shock Delivered	10.51 (9.17–12.05)	<0.001	3.01 (2.57–3.53)	<0.001

**Initial Cardiac Rhythm**	**Initial Rhythm**	*Asystole*	*Ref.*	*–*	*–*	*–*
		Bradycardia	4.52 (3.92–5.22)	<0.001	3.54 (3.01–4.17)	<0.001
		PEA	3.38 (3.22–3.54)	<0.001	2.28 (2.17–2.41)	<0.001
		VF/VT	6.22 (5.93–6.52)	<0.001	4.50 (4.27–4.75)	<0.001

**EMS Variables**	**EMS Total Response Time**	*≤8 min*	*Ref.*	*–*	*–*	*–*
		8–15 min	0.72 (0.69–0.75)	<0.001	0.75 (0.72–0.79)	<0.001
		>15 min	0.53 (0.50–0.56)	<0.001	0.69 (0.66–0.73)	<0.001
	**Advanced Airway Use**	*Advanced Airway*	*Ref.*	*–*	*–*	*–*
		No Advanced Airway	0.09 (0.09–0.10)	<0.001	0.17 (0.16–0.18)	<0.001
	**Mechanical Chest Compression**	*No*	*Ref.*	*–*	*–*	*–*
		Yes	1.64 (1.57–1.70)	<0.001	0.89 (0.85–0.93)	<0.001

Unadjusted (univariable) and adjusted (multivarible) odds ratios (OR) with 95% confidence intervals (CI) for independent associations with survival in the total cohort. Variables include patient demographics (age, sex), urbanisation level (urban vs. rural), arrest location (home vs. public), bystander intervention, initial cardiac rhythm assessed by EMS, and EMS response time and time of the day of OHCA.

In the multivariable analysis, rural location remained independently associated with lower survival odds (OR = 0.83, 95 % CI 0.79–0.87, p < 0.001) after comprehensive adjustment for all other variables in the multivariable model (Table 1). VF/VT had the highest adjusted odds ratio for survival among all types of rhythms (OR = 4.50, 95 % CI 4.27–4.75), and longer EMS response times showed a time-dependent relationship with worse outcomes (8–15 min: OR = 0.75, 95 % CI 0.72–0.79; >15 min: OR = 0.69, 95 % CI 0.66–0.73; p < 0.001 for all). After adjustment, age > 65 years was associated with lower survival (OR = 0.64, 95 % CI 0.61–0.67, p < 0.001). Female sex was positively associated with survival in the adjusted model (OR = 1.22, 95 % CI 1.17–1.27, p < 0.001), contrasting with the negative effect shown by the univariable analysis. After adjustment for other variables, mechanical chest compression showed a reversed association in the multivariable model (OR = 0.89, 95 % CI 0.85–0.93, p < 0.001). Survival was lower for home arrests (OR 0.55, 95 % CI 0.52–0.57) and other locations (OR 0.68, 95 % CI 0.63–0.74) compared to public locations. Relative to cases occuring between 0 and 8 h, calls at 8–16 h (OR 1.07, 95 % CI 1.01–1.12) and 16–24 h (OR 1.08, 95 % CI 1.03–1.15) had slightly higher odds of ROSC.

Examining intervention effects across settings, mechanical chest compression devices were employed in a similar proportion of cases across urban (32.7 %) and rural (31.5 %) cases, its usage showed significant positive associations with survival in univariable analysis overall (OR = 1.64, 95 % CI 1.57–1.70, p < 0.001), with consistent effects in both urban (OR = 1.69, 95 % CI 1.62–1.77, p < 0.001) and rural settings (OR = 1.49, 95 % CI 1.38–1.60, p < 0.001) (Supplementary Tables 3 and 4).

Our analysis of Model-Based Variable Influence ranked the initial cardiac rhythm as the factor with the highest relative importance for survival (100 %), followed by bystander-witnessed status (78.3 %), lower EMS response time (56.1 %), and younger age (42.7 %). Assessment of multicollinearity confirmed VIF values <10 for all factors (Supplementary Table 5).

### Propensity score matching analysis

**Fig. 1:**
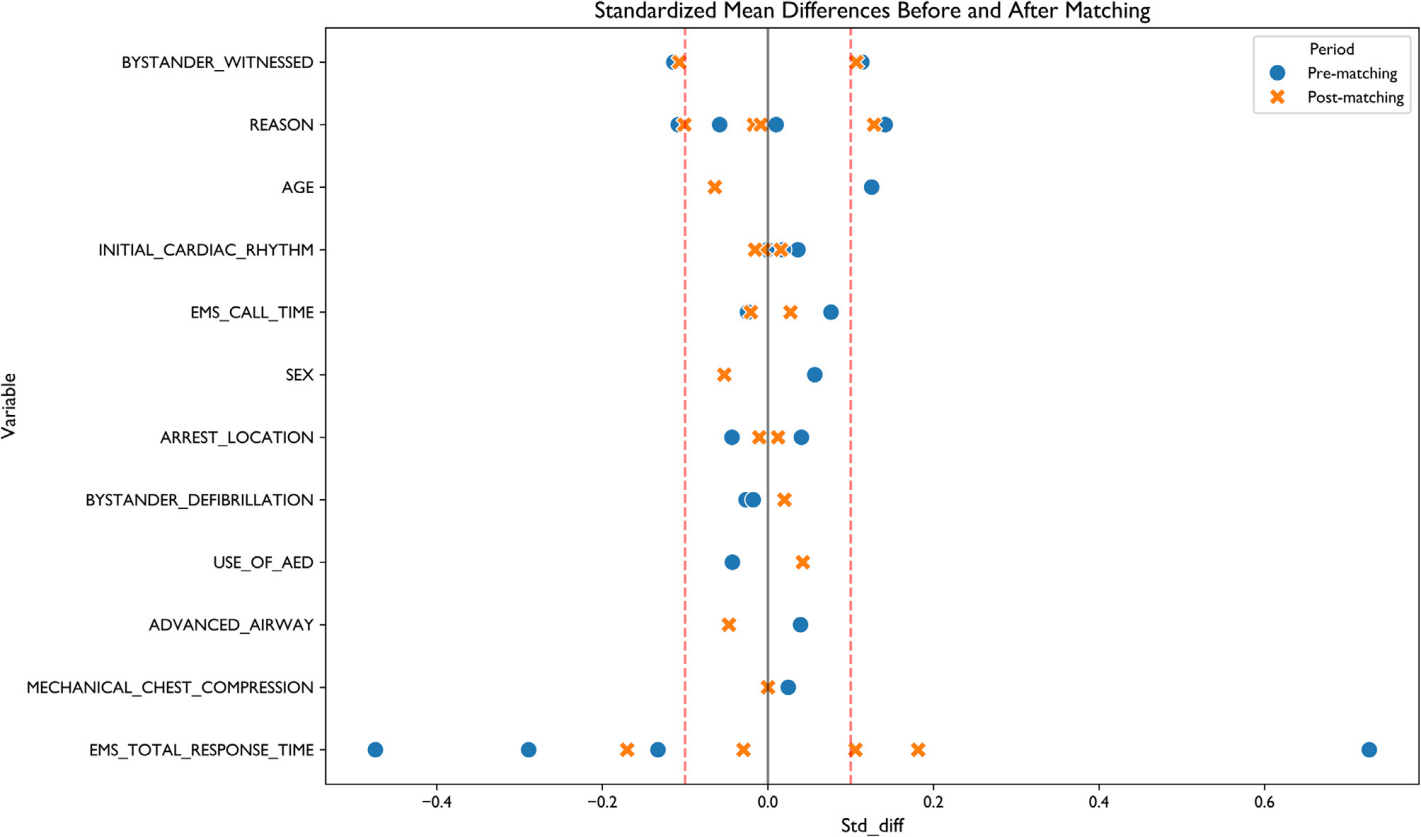
**Standardised Mean Differences for Baseline Characteristics Before and After Matching in Urban and Rural OHCA Cases.** The love plot presents the standardised mean differences (SMD) for key baseline characteristics before and after propensity score matching in urban and rural OHCA cohorts. Before matching, substantial imbalances were observed in multiple variables, including age, bystander-witnessed status, and EMS response times, reflecting marked demographic and structural differences between urban and rural cases. The dashed lines at ±0.1 represent the common threshold for acceptable balance after matching. Following matching, the balance of covariates improved substantially, with most SMDs reduced below the 0.1 threshold, indicating successful adjustment for baseline disparities. EMS response time showed residual imbalance after matching. This confirms that the matched cohorts were sufficiently comparable to isolate the independent effect of location on outcomes.

**Supplementary Fig. 1:**
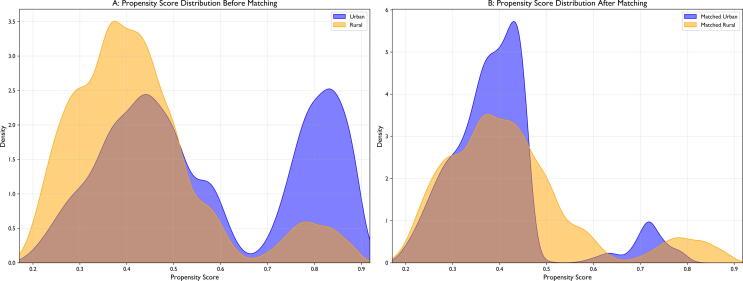


The matching process successfully paired 38,734 urban cases with 38,734 rural cases (99.5 % matching rate), with standardised mean differences for key covariates reduced from a pre-matching range of 0.01–0.73 to a post-matching range where all values were below 0.1, confirming adequate balance (Fig. 1) (Supplementary Table 6). Propensity score distributions before and after matching demonstrated excellent overlap and balance between urban and rural groups (Supplementary Fig. 1).

In the matched cohort, urban location remained significantly associated with higher survival (10.28 % vs rural 8.36 %, p < 0.001; OR = 1.26, 95 % CI 1.20–1.32). The absolute difference in survival rates between urban and rural cases was 1.92 %.

### Continuous response time analysis

Sensitivity analysis using natural cubic splines revealed a steep decline in survival probability with increasing response time and demonstrated significant rural × time interaction (likelihood-ratio test χ^2^ = 54.08, df = 6, p < 0.001). The interaction confirms that urban–rural survival differences vary systematically across the response time spectrum rather than being uniform (Fig. 3). The urban advantage was most pronounced at shortest response times (≤5 min: OR = 2.37, 95 % CI 1.87–3.00) and decreased as response times increased, with rural disadvantage primarily affecting time-critical interventions (Supplementary Fig. 5).

### Model performance and validation

**Fig. 2:**
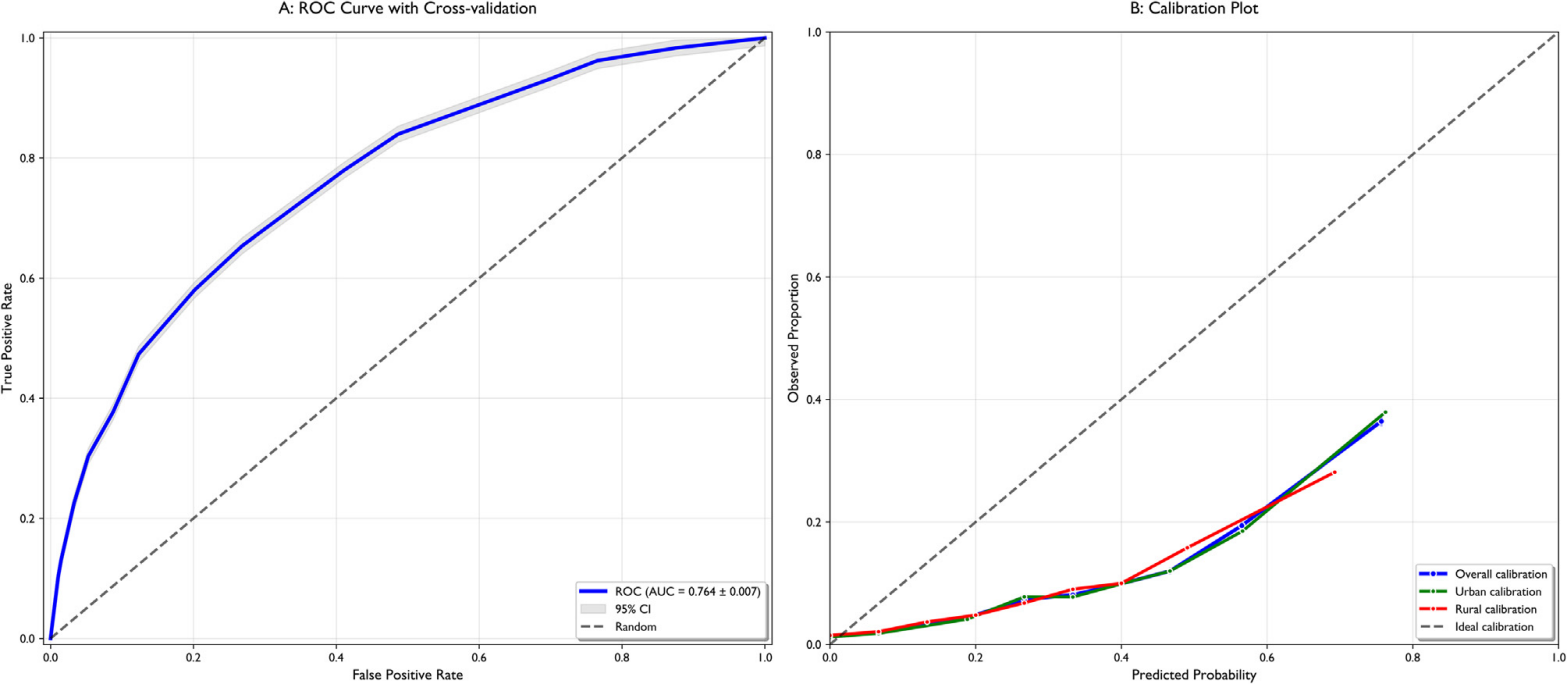
**Model Performance and Calibration in the Matched Cohort.** (A) Receiver operating characteristic (ROC) curve demonstrating the discriminatory ability of the model in the matched cohort (AUC = 0.764). The blue line represents the ROC curve, with the grey shaded area showing the 95 % confidence interval. (B) The calibration plot compares the predicted versus observed survival probabilities, showing the relationship between model estimates and actual outcomes across urban (green), rural (red), and overall cohort (blue) predictions. The deviation from the ideal diagonal line (dashed) indicates some model miscalibration, particularly in the higher probability ranges (>0.7) where predictions slightly overestimate actual outcomes. Despite this visual difference, the overall calibration remains statistically acceptable as confirmed by the non-significant result of the Hosmer-Lemeshow test (p = 0.218). This pattern of calibration is common in rare-event prediction models and indicates that predicted probabilities may require minor adjustment for probabilities exceeding 0.7. (For interpretation of the references to colour in this figure legend, the reader is referred to the web version of this article.)

**Supplementary Fig. 2:**
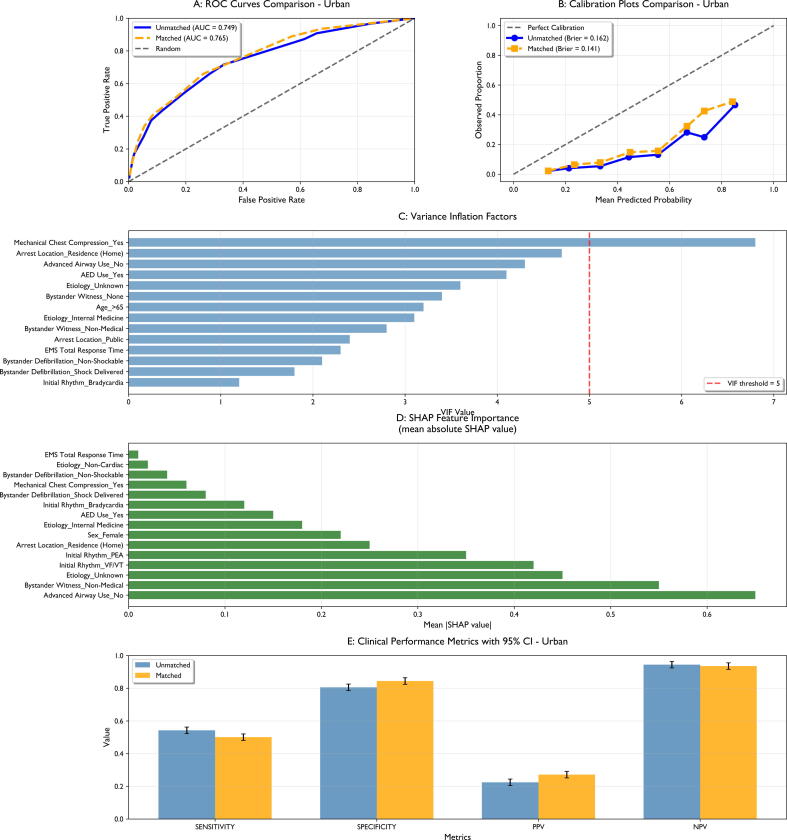


**Supplementary Fig. 3:**
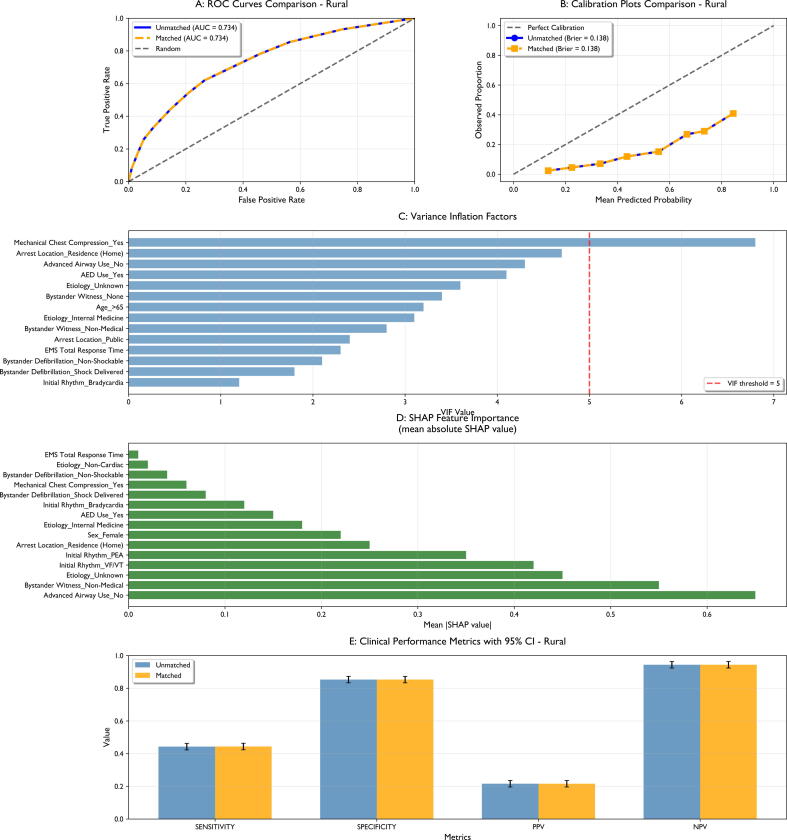


**Supplementary Fig. 4:**
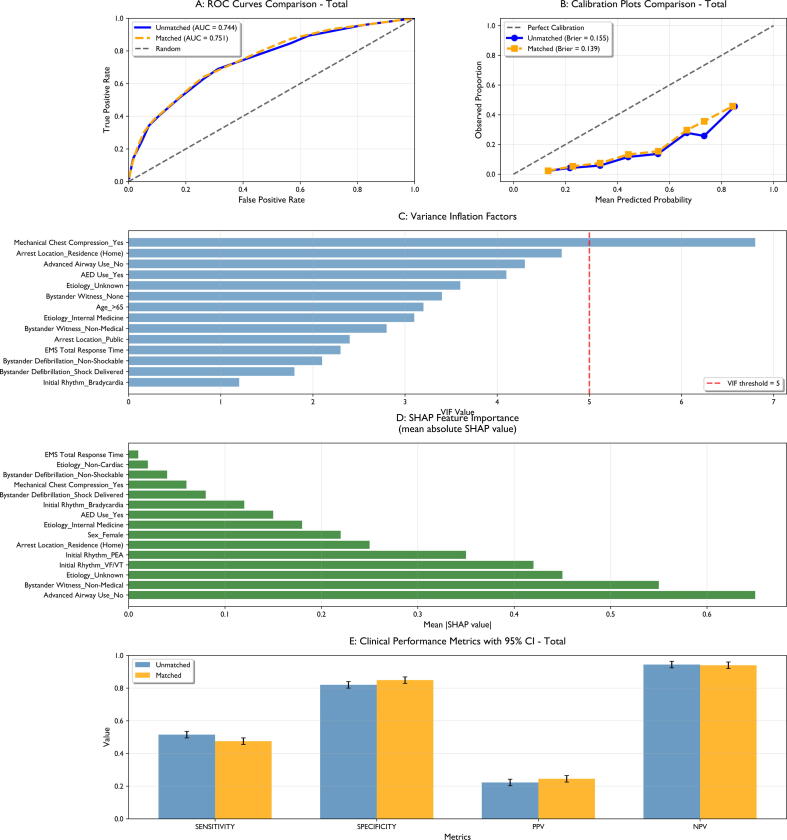


The multivariable model demonstrated excellent discrimination in assessing survival odds with a cross-validation AUC of 0.856 (95 % CI 0.853–0.860) for the unmatched dataset and 0.845 (95 % CI 0.842–0.850) for the matched cohort (Fig. 2). A more conservative bootstrap validation yielded an optimism-corrected AUC of 0.764 (95 % CI 0.760–0.769), suggesting robust generalizability with minimal overfitting (optimism of only 0.00009) (Supplementary Table 7). Model performance remained consistent across urban and rural subgroups, supporting the robustness of the findings (Supplementary Figs. 2 and 3). A non-significant result of the Hosmer-Lemeshow test confirmed the model's exact calibration (p = 0.218). Detailed validation analyses, including ROC curves, calibration plots, variance inflation factors, and SHAP feature importance analyses for urban, rural, and total populations, are presented in Supplementary Figs. 2 and 4, and Supplementary Table 8, respectively.

**Fig. 3:**
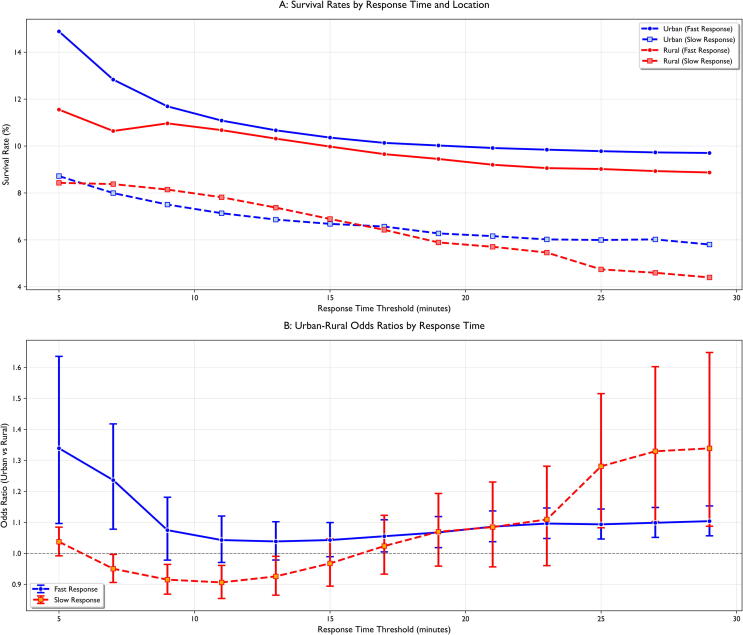
**Impact of EMS Response Time Thresholds on Urban-Rural Survival Disparities. (A) Survival Rates**. This panel demonstrates how survival rates differ between urban and rural settings across various EMS response time thresholds. Each threshold on the x-axis (5, 8, 10, 15, 20, and 30 min) creates two patient groups: those with 'fast' responses (≤ threshold, solid lines) and those with 'slow' responses (>threshold, dashed lines). The y-axis shows survival percentages. For urban settings (blue lines), patients receiving fast responses (solid blue) consistently show higher survival rates (10–23 %) compared to those with delayed responses (dashed blue, 7–10 %). Similarly, for rural settings (red lines), fast responses (solid red) yield better outcomes (8–12 %) than delayed responses (dashed red, 4–8 %). Notably, the survival advantage of fast response is more pronounced in urban areas, particularly at the earliest time points (≤5 min), where urban survival reaches approximately 23 % compared to 12 % in rural areas. **(B) Urban-Rural Odds Ratios**. This panel quantifies the urban survival advantage relative to rural areas, expressed as odds ratios (OR). The blue line represents the urban–rural OR for patients with 'fast' responses, whereas the orange line shows this relationship for 'slow' responses. For fast responses (blue line), we observe that at the 5-min threshold, urban patients have 2.37 times higher odds of survival than rural patients (OR = 2.37, 95 % CI 1.87–3.00). This advantage gradually diminishes as the threshold increases, reaching OR = 1.22 (95 % CI 1.15–1.31) at the 30-min threshold. For slow responses (orange line), a different pattern emerges, at early thresholds, the urban–rural difference is minimal (OR = 1.20 at 5 min), the urban advantage grows substantially at longer thresholds, reaching OR = 2.13 (95 % CI 1.78–2.54) at 30 min. These contrasting patterns suggest that urban settings maintain an advantage throughout all response times, but the nature of this advantage changes. For rapid responses, urban advantage likely stems from factors such as more experienced providers and better system coordination. For delayed responses, the advantage may reflect greater availability of advanced life support resources in urban areas that can sustain resuscitation efforts over longer periods. (For interpretation of the references to colour in this figure legend, the reader is referred to the web version of this article.)

Sensitivity analyses using varying caliper widths (0.1 to 0.25) and alternative model specifications yielded comparable results. The AUC varied slightly across different caliper settings, ranging from 0.842 to 0.850, indicating stability in the model performance.

### Response time threshold analysis

Analysis of different EMS response time thresholds revealed that the urban survival advantage was most pronounced in cases with response times ≤ 8 min (13.2 % vs rural: 8.7 %, p < 0.001, OR = 1.59, 95 % CI 1.44–1.76) (Fig. 3). This difference decreased but remained significant at 8–15 min (10.3 % vs rural: 9.1 %, p = 0.002, OR = 1.14, 95 % CI 1.05–1.25), and was non-significantly attenuated further with response times >15 min (8.2 % vs rural: 7.8 %, p = 0.067, OR = 1.06, 95 % CI 0.99–1.13).

The nature of the urban–rural disparity varied with response timing: for fast responses (≤threshold), the urban advantage for survival was strongest at early thresholds (OR at 5 min = 2.37, 95 % CI 1.87–3.00) and decreased as thresholds increased; in contrast, for slow response times (>threshold), the urban advantage increased substantially at higher thresholds (OR at 30 min = 2.13, 95 % CI 1.78–2.54).

### Subgroup and sensitivity analyses

**Fig. 4:**
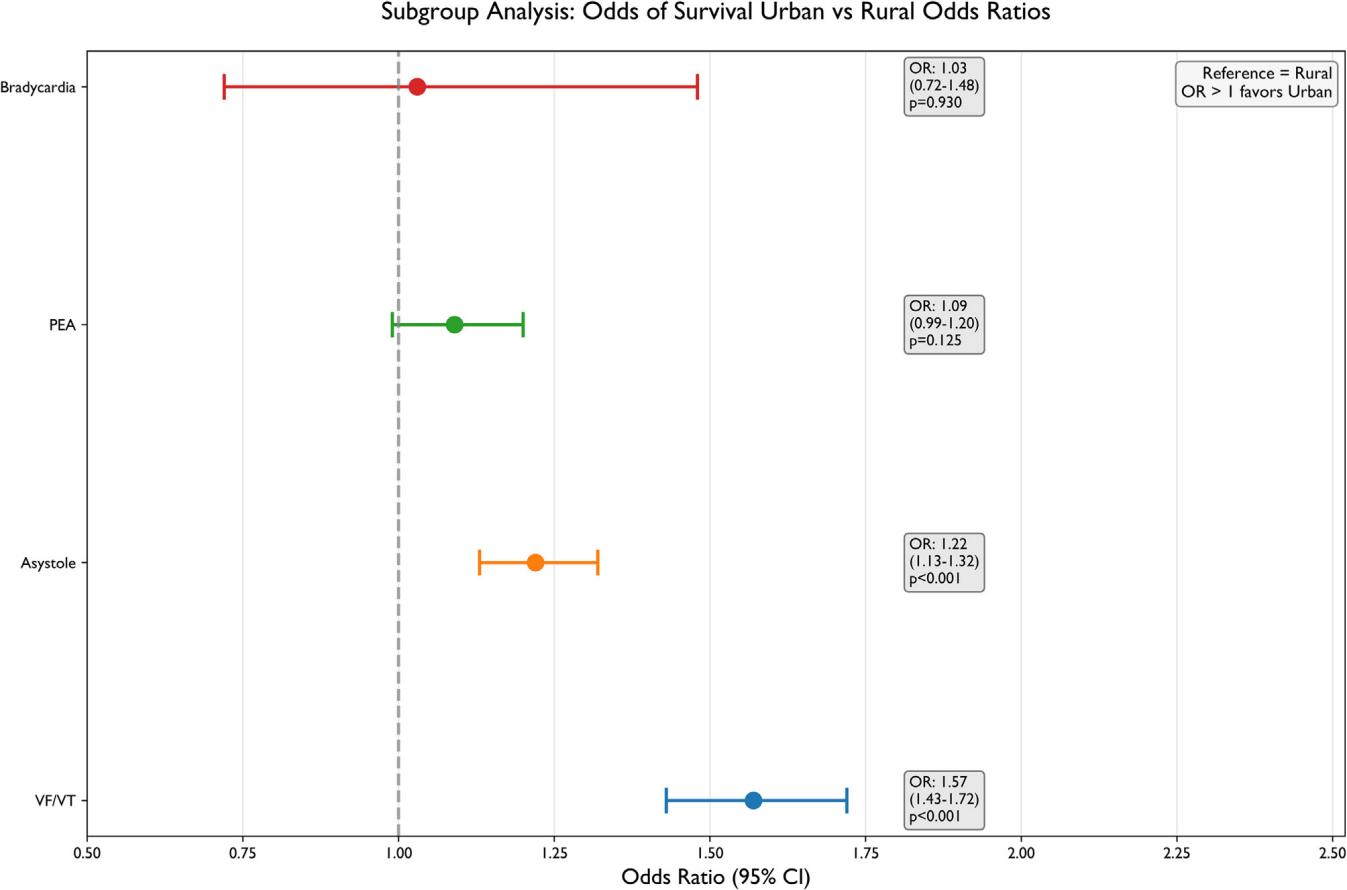
**Subgroup Analysis: Urban vs Rural Odds Ratios.** Forest plot showing the odds ratios with 95 % confidence intervals for urban versus rural survival across different initial cardiac rhythms. The vertical dashed line at OR = 1.0 represents equal odds between settings, whereas OR > 1.0 represents better survival of urban-located OHCA. The highest urban advantage was observed in VF/VT (OR = 1.57) and bradycardia (OR = 1.03), whereas less pronounced in asystole (OR = 1.22) and PEA (OR = 1.09). Odds ratios with corresponding 95 % confidence intervals and p-values are shown on the right side.

Urban-rural survival disparities varied significantly across clinical subgroups. The urban survival advantage was most pronounced for shockable rhythms (VF/VT: OR = 1.57, 95 % CI 1.43–1.72, p < 0.001) (Fig. 4) and cardiac etiology (OR = 1.35, 95 % CI 1.26–1.46, p < 0.001), while being smaller but still significant in asystole (OR = 1.22, 95 % CI 1.13–1.32, p < 0.001) (Table 2). The urban survival advantage was significantly stronger for witnessed cardiac arrests (OR = 1.31, 95 % CI 1.20–1.42, p < 0.001), whereas no significant urban–rural difference was observed for unwitnessed arrests (OR = 1.00, 95 % CI 0.89–1.13, p = 1.00) (Table 2).

**Table 2:** Sensitivity Analysis Results by Subgroup.

**Subgroup (Variable)**	**Subgroup Level**	**N_total**	**N_urban**	**N_rural**	**Urban Survival (%)**	**Rural Survival (%)**	**Survival Diff (%)**	**Odds Ratio (95 % CI)**	**p-value**
**Initial Cardiac Rhythm**	**VF/VT**	9166	4662	4504	30.2	21.63	8.58	1.57 (1.43–1.72)	<0.001
	**PEA**	12,354	6095	6259	15.93	14.83	1.1	1.09 (0.99–1.20)	0.12
	**Asystole**	55,193	27,602	27,591	5.53	4.58	0.95	1.22 (1.13–1.32)	<0.001
	**Bradycardia**	755	375	380	20.27	19.74	0.53	1.03 (0.72–1.48)	0.93

**Cardiac Arrest Reason (Etiology)**	**Cardiac**	21,356	11,386	9970	17.81	13.79	4.02	1.35 (1.26–1.46)	<0.001
	**Non-Cardiac**	4698	2342	2356	13.19	12.95	0.25	1.02 (0.86–1.21)	0.85
	**Internal Disease**	23,262	11,838	11,424	10.74	10.65	0.08	1.01 (0.93–1.10)	0.85
	**Unknown**	28,152	13,168	14,984	2.84	2.29	0.55	1.25 (1.08–1.45)	0.01

**Bystander Witness**	**Medical-witnessed**	15,941	8623	7318	18.43	14.73	3.7	1.31 (1.20–1.42)	<0.001
	**Non-Medical**	32,263	16,706	15,557	11.23	9.97	1.26	1.14 (1.06–1.23)	<0.001
	**None**	29,264	13,405	15,859	3.86	3.85	0	1.00 (0.89–1.13)	1.00

**Age**	**≤ 65**	32,518	17,100	15,418	13.42	10.68	2.74	1.30 (1.21–1.39)	<0.001
	**> 65**	44,950	21,634	23,316	7.8	6.83	0.97	1.15 (1.07–1.24)	<0.001

Survival odds ratios across different subgroups, including age, initial cardiac rhythm, and EMS response time, were assessed to indicate the robustness of the primary findings. Adjusted odds ratios and confidence intervals are reported.

## Discussion

Our analysis of 130,258 OHCA cases demonstrates persistent urban–rural survival disparities in Hungary, with rural location independently associated with lower survival odds (OR = 0.83, 95 % CI 0.79–0.87) even after comprehensive propensity score matching.

Our analysis employed several methodological refinements to address potential confounding. Continuous response time modeling using natural cubic splines revealed significant interaction effects, suggesting that urban–rural disparities are not uniform across all emergency response scenarios but vary systematically with response time.

Our findings align with international literature documenting urban–rural OHCA disparities, though effect magnitudes vary across healthcare systems. Consistent with findings from Irish, Danish, and Swedish national registries, we observed significant rural disadvantage, though our urban–rural odds ratio of 1.26 is smaller than the two-fold survival difference observed in the Irish registry. This smaller effect likely reflects Hungary's unified national EMS system, which standardizes protocols, equipment, and training across all regions, potentially reducing system-level disparities that contribute to larger differences in countries with fragmented EMS services.^9^, ^29^, ^30^, ^31^, ^32^, ^33^

As noted in other studies, the rural disadvantage is particularly pronounced for patients with an initial shockable rhythm. This is because longer EMS response times in rural areas increase the likelihood that these time-sensitive rhythms will deteriorate into non-shockable, less treatable states before defibrillation can be administered.^29^, ^34^, ^35^, ^36^ Our analysis identified VF/VT as the initial cardiac rhythm to be the strongest factor associated with survival (OR: 4.95 vs asystole as reference), followed by bystander-witnessed status (OR: 2.08 vs no witness) and faster EMS response time (OR: 1.45 for ≤ 8 min vs > 15 min). These findings are consistent with international predictive models that identify shockable rhythm as a dominant positive factor, associated with 4–6 times higher survival odds than asystole.^37^, ^38^, ^39^ After adjusting for the factors of initial cardiac rhythm and EMS response time, rural location remained independently associated with worse survival (OR = 0.83), suggesting that unmeasured or undetected variables may contribute to the disparity.

Bystander defibrillation with successful shock delivery was strongly associated with survival, emphasizing the operational importance of effective shock delivery when indicated. In rural settings with longer EMS response times, strategies to increase both AED deployment and effective CPR and appropriate shock delivery could significantly reduce the urban–rural survival gap. Public access defibrillation programs should therefore emphasise both strategic AED placement and effective training in their use.

The similar deployment rates of mechanical chest compression devices in urban and rural settings demonstrate equitable resource allocation, potentially mitigating other aspects of the urban–rural disparity. Our analysis revealed an important pattern regarding mechanical chest compression devices. Hungary has implemented one of Europe's most comprehensive nationwide mechanical chest compression programs, with LUCAS™ devices deployed strategically according to unified protocols across EMS units in both urban and rural settings. Whereas the univariable analysis showed a positive association with survival, multivariable adjustment revealed an inverse relationship, likely reflecting confounding by indication, as these devices are typically used in more complex resuscitation cases with a potential poorer prognosis. Despite this statistical finding, mechanical devices serve a critical function by maintaining consistent, high-quality compressions during transport, particularly valuable in rural settings with longer transport distances. Additionally, during onsite advanced life support, the use of mechanical chest compression devices can free up one or two rescuers who would otherwise be dedicated to manual compressions, allowing them to perform other essential resuscitation tasks more rapidly, such as establishing airway access, administering medications, or operating the defibrillator. Future research should investigate the optimal timing of device deployment and identify patient subgroups who benefit most from this intervention.

It is important to note that the Hungarian National Ambulance Service represents a unique, unified nationwide emergency medical system,^13^ unlike the fragmented local or regional services described in many comparative studies.^18^, ^25^, ^34^, ^40^ This organisational structure allows for standardized protocols, centralized dispatcher teams who allocate the units to cases, training, and equipment deployment across both urban and rural settings. Additionally, response time measurements in our system follow consistent national protocols, which may differ from metrics used in other studies. This context should be considered when interpreting our findings in relation to international literature on urban–rural disparities.

After propensity score matching, we analysed urban–rural survival differences within matched response time categories. Modeling response time as a continuous variable with natural cubic splines showed a clear, dose-dependent decline in survival as delays increased, consistent with prior work estimating that each minute beyond 5 min reduces the odds of favorable outcomes by approximately 8 % in the absence of bystander CPR.^41^, ^42^, ^43^, ^44^, ^45^ Based on our results, the urban advantage was concentrated at short response times, with the strongest difference at ≤8 min (OR = 1.59) and attenuation across 8–15 min, becoming minimal at longer delays. A significant rural × time interaction confirms that the urban–rural gap varies across the time spectrum and is most pronounced when early links of the Chain of Survival are achieved rapidly. The unexpected finding of higher rural survival in the 8–15 min response category (9.8 % vs 8.5 % urban, p < 0.001) requires careful interpretation. Several factors may contribute to this paradox: (1) Geographic selection bias—rural cases with intermediate response times may represent more accessible locations with better baseline characteristics compared to urban cases requiring similar transport times, which may include more complex logistical challenges; (2) Different case mix—rural intermediate-response cases may have higher proportions of witnessed arrests or medical etiology; (3) EMS deployment strategies may differ between settings during this time window. However, this finding should be interpreted cautiously as it contrasts with the overall rural disadvantage observed at both shorter (≤8 min) and longer (>15 min) response times, and requires validation in datasets with more detailed geographic and clinical variables. Our threshold analysis revealed that even when adjusting for patient characteristics and response times, urban survival advantage remained significant across different time thresholds. For cases with rapid response (≤5 min), the urban survival advantage was substantial (OR = 2.37). This advantage decreased but remained statistically significant with longer response times (OR = 1.22, for >5 min).

Additionally, our post-matching analysis shows that despite the successful balancing of most covariates, response time distribution differed slightly between urban and rural groups. Urban areas had a higher proportion of cases with extended response times (>15 min: 55.1 % vs 49.0 %), yet still maintained superior survival outcomes (10.3 % vs 8.4 %, p < 0.001), suggesting that the urban areas have more response calls, but a consecutively higher success rate when reacting. This suggests that factors beyond response time alone, such as differences in bystander CPR quality, EMS provider experience, contribute to the adjusted urban survival advantage.

Public Access Defibrillation programs serve as critical interventions that can significantly influence survival outcomes independent of EMS arrival times, particularly for shockable rhythms. Strategic AED placement in rural communities, bystander first responder education, bystander CPR schemes, optimized dispatch algorithms, telecommunicator-guided CPR, and innovative solutions like drone-delivered AEDs are the essential components of such programmes.^46^, ^47^, ^48^, ^49^ Our findings support the development of integrated community response systems that strengthen the Chain of Survival in rural areas, where well-implemented PAD initiatives can significantly mitigate the survival disadvantage caused by inevitably longer EMS response times.

Future research should explore the implementation and cost-effectiveness of these interventions across different rural contexts. Additionally, investigation of hospital-level factors contributing to the urban–rural disparities after initial resuscitation is warranted, as our study was limited to ROSC on-scene without the availability of long-term outcome data.

### Limitations

Several limitations should be acknowledged. First, our binary urban–rural classification, while based on official administrative categories, does not capture the full spectrum of geographic variation within these broad categories. More granulated analysis distinguishing between metropolitan areas, small cities, large villages, and remote rural areas might reveal additional nuances in outcome patterns and identify specific geographic thresholds where disparities emerge. Second, our analysis was limited to on-scene ROSC without access to other critical survival outcomes including survival to hospital admission, survival to hospital discharge, and long-term neurological outcomes, which may not fully represent the clinical impact of urban–rural disparities.

Third, etiology classification maintains “unknown” as a separate category; while this preserves clinical granularity, it differs from current Utstein recommendations that suggest grouping unknown cases as presumed cardiac. Fourth, unmeasured confounding factors such as community-level socioeconomic characteristics, regional variations in bystander response training, or hospital-level factors following initial resuscitation may influence observed disparities. Finally, while all EMS crews have standardized advanced life support training, individual crew experience and performance metrics were not available in our dataset, potentially representing unmeasured confounding factors that could vary between urban and rural settings.

## Conclusion

Our large-scale analysis of 130,258 OHCA cases represents a comprehensive nationwide assessment of urban–rural disparities in OHCA outcomes in Hungary using propensity score methodology. Rural location was independently associated with lower survival (OR = 0.83). The largest potential gains were observed for VF/VT arrests and medical-witnessed cases, highlighting targets for organizational improvement in rural communities. We demonstrated that significant differences in survival persist even after thorough adjustment for patient, cardiac arrest, and treatment characteristics. Urban location was independently associated with higher survival, with the advantage most pronounced in patients with shockable rhythms, witnessed arrests, and proven cardiac aetiology. While EMS response times were substantially longer in rural areas, these differences only partially explained the observed disparities. Continuous response time modeling revealed significant interaction effects, indicating that disparities vary across the emergency response spectrum. These findings highlight the need for targeted interventions addressing both response time optimization and system-level factors in rural communities. The presence of a nationally uniform, protocol-driven ambulance service with homogeneous equipment and highly trained staff represents a unique strength, offering a solid foundation for further system-level improvements and quality initiatives.

## Declaration of Generative AI and AI-assisted technologies in the writing process

During the preparation of this work, the author(s) used Claude Sonnet 3.7, ChatGPT, and Grammarly to enhance language clarity and improve grammar and style. After using these tools, the authors reviewed and edited the content as needed and take full responsibility for the content of the publication.

## CRediT authorship contribution statement

**Ádám Pál-Jakab:** Writing – review & editing, Writing – original draft, Visualization, Validation, Methodology, Data curation, Conceptualization. **Bettina Nagy:** Writing – review & editing, Validation, Methodology. **Boldizsár Kiss:** Writing – review & editing, Data curation, Conceptualization. **György Pápai:** Writing – review & editing, Resources, Investigation. **Nora Boussoussou:** Resources, Investigation. **Béla Merkely:** Resources, Project administration, Funding acquisition. **Miklós Constantinovits:** Writing – review & editing, Resources, Investigation. **Gábor Csató:** Writing – review & editing, Resources, Investigation. **Péter Sótonyi:** Resources, Investigation. **Brigitta Szilágyi:** Visualization, Validation, Methodology, Funding acquisition, Data curation. **Endre Zima:** Writing – review & editing, Validation, Resources, Project administration, Methodology, Investigation, Funding acquisition, Conceptualization.

## Funding

Open-access funding is provided by 10.13039/501100002332Semmelweis University. The study was supported by the PhD Excellence Program of Semmelweis University by grant EFOP-3.6.3-VEKOP-16-2017-00009 (“Semmelweis 250 + Excellence Scholarship”). Project no. RRF-2.3.1-21-2022-00014 has been implemented with the support provided by the European Union within the Hungarian Climate Change National Laboratory. This project was supported by RRF-2.3.1-21-2022-00003, within the framework of the National Cardiovascular Laboratory Artificial Intelligence Core Lab. B.M. reports institutional grants to the Heart and Vascular Center of Semmelweis University from Biotronik, Boehringer Ingelheim, DUKE Clinical Institute, Eli Lilly, and Novartis, and personal fees from Boehringer Ingelheim, Daiichi Sankyo, DUKE Clinical Institute, and Novartis, all outside the submitted work. B.S. contributed to project no. TKP2021-EGA-02, implemented with the support provided by the Ministry of Culture and Innovation of Hungary from the National Research, Development and Innovation Fund, financed under the TKP2021-EGA funding scheme. E.Z. reports personal fees from Biotronik, Abbott, AstraZeneca, Innomed, Boston Scientific, and Medtronic, all outside the submitted work. The funders had no role in the design of the study; in the collection, analyses, or interpretation of data; in the writing of the manuscript; or in the decision to publish the results.

## Declaration of competing interest

The authors declare that they have no known competing financial interests or personal relationships that could have appeared to influence the work reported in this paper.

## Figures and Tables

**Supplementary Fig. 5:**