# Supplementary material for: Urban-rural disparities in out-of-hospital cardiac arrest outcomes: a nationwide Hungarian study
Source: Resusc Plus. 2025 Sep 23;26:101108. doi: 10.1016/j.resplu.2025.101108 (PMC12546963; doi:10.1016/j.resplu.2025.101108)
Supplement: Supplementary Data 6 [file mmc6.doc]

**Urban-Rural Disparities in Out-of-Hospital Cardiac Arrest Outcomes: A Nationwide Hungarian Study**

**Supplementary Materials**

1. Supplementary Figure 1: Propensity Score Distributions Before and After Matching

2. Supplementary Figure 2: Urban Model Validation

3. Supplementary Figure 3: Rural Model Validation

4. Supplementary Figure 4: Total Cohort Model Validation

5. Supplementary Figure 5: Adjusted Probability of ROSC by EMS Response Time Using Natural Cubic Splines

6. Supplementary Table 1: Baseline Characteristics of the Study Population

7. Supplementary Table 2: Detailed Survival Outcomes by Patient Characteristics

8. Supplementary Table 3: Urban Subgroup Predictors of Survival

9. Supplementary Table 4: Rural Subgroup Predictors of Survival

10. Supplementary Table 5: Variance Inflation Factors

11. Supplementary Table 6: Covariate Balance Before and After Matching

12. Supplementary Table 7: Cross-Validation Results

13. Supplementary Table 8: Propensity Score Model Coefficients

**Supplementary Materials - Figures**

**Supplementary Figure 1. Propensity Score Distributions Before and After Matching**

(A) The density plot shows the distribution of propensity scores before matching, with urban (blue) and rural (orange) populations. The substantial separation between distributions indicates significant differences in baseline characteristics between urban and rural cases before matching. (B) The density plot shows the distribution of propensity scores after matching, with urban (blue) and rural (orange) populations. The improved overlap between distributions demonstrates successful balancing of covariates through the matching process.

**Supplementary Figure 2. Urban Model Validation**

(A) Receiver operating characteristic (ROC) curves illustrate the comparison of model discrimination between matched and unmatched urban cohorts (AUC = 0.772 and 0.769, respectively). (B) Calibration plots demonstrate the agreement between predicted and observed probabilities for matched and unmatched cohorts. The Brier scores (unmatched: 0.134, matched: 0.126) quantify overall prediction accuracy, with lower values indicating better calibration (0 = perfect prediction, 0.25 = non-informative model for binary outcomes). The slightly higher Brier score in the matched cohort suggests minimal calibration cost from the matching process while maintaining good predictive performance. (C) Variance inflation factors (VIF) indicate multicollinearity among predictors included in the model; the red dashed line marks the commonly used threshold of VIF = 5. (D) SHapley Additive exPlanations (SHAP) feature importance values depict the relative contributions of predictors to the model. (E) Clinical performance metrics (sensitivity, specificity, positive predictive value [PPV], and negative predictive value [NPV]) are presented with their corresponding 95% confidence intervals for matched and unmatched urban cohorts. Abbreviations: AUC, area under the curve; CI, confidence interval; NPV, negative predictive value; PPV, positive predictive value; ROC, receiver operating characteristic; SHAP, SHapley Additive exPlanations; VIF, variance inflation factor.

**Supplementary Figure 3. Rural Model Validation**

(A) ROC curves comparing model discrimination in matched versus unmatched rural cohorts (AUC = 0.750 and 0.750, respectively). (B) Calibration plots demonstrating agreement between predicted and observed probabilities. The Brier scores (both unmatched and matched: 0.120) quantify overall prediction accuracy, with lower values indicating better calibration (0 = perfect prediction, 0.25 = non-informative model for binary outcomes). (C) Variance inflation factors for predictors used in the model; red dashed line indicates VIF threshold of 5. (D) SHAP feature importance values indicating the relative contribution of each predictor to the model. (E) Clinical performance metrics displaying sensitivity, specificity, PPV, and NPV with 95% confidence intervals for matched and unmatched cohorts. Abbreviations: AUC, area under the curve; CI, confidence interval; NPV, negative predictive value; PPV, positive predictive value; ROC, receiver operating characteristic; SHAP, SHapley Additive exPlanations; VIF, variance inflation factor.

**Supplementary Figure 4. Total Cohort Model Validation**

(A) ROC curves comparing model discrimination in matched versus unmatched total cohorts (AUC = 0.763 and 0.764, respectively). (B) Calibration plots demonstrating agreement between predicted and observed probabilities. The Brier scores (unmatched: 0.130, matched: 0.123) quantify overall prediction accuracy, with lower values indicating better calibration (0 = perfect prediction, 0.25 = non-informative model for binary outcomes). The slight increase in Brier score for the matched cohort indicates minimal calibration cost from the matching process while maintaining good predictive performance. (C) Variance inflation factors for predictors used in the model; red dashed line indicates VIF threshold of 5. (D) SHAP feature importance values indicate the relative contribution of each predictor to the model. (E) Clinical performance metrics displaying sensitivity, specificity, PPV, and NPV with 95% confidence intervals for matched and unmatched cohorts. Abbreviations: AUC, area under the curve; CI, confidence interval; NPV, negative predictive value; PPV, positive predictive value; ROC, receiver operating characteristic; SHAP, SHapley Additive exPlanations; VIF, variance inflation factor.

**Supplementary Figure 5. Adjusted Probability of ROSC by EMS Response Time Using Natural Cubic Splines**

Adjusted survival probability curves stratified by urban and rural location, modeled using natural cubic splines (degrees of freedom=3) with 95% confidence intervals. The curves demonstrate the dose-dependent decline in survival probability with increasing EMS response time in both settings. The rural×response time interaction was statistically significant (likelihood-ratio test χ² = 54.08, degrees of freedom = 6, p < 0.001), indicating that urban-rural survival differences vary across the response time spectrum. The urban survival advantage is most pronounced at shorter response times and gradually attenuates with longer delays. Confidence intervals represent uncertainty in the spline predictions across the response time continuum.

**Supplementary materials - Tables**

Supplementary Table 1. Baseline Characteristics of the Study Population

| **Category** | **Variable** | **Total** | **Urban** | **Rural** | **p-value** |
| --- | --- | --- | --- | --- | --- |
| **Bystander Variables** | **Bystander Defibrillation** | No: 127378 (97.80%) Non-Shockable Rhythm: 2038 (1.60%) Shock Delivered: 842 (0.60%) | No: 89452 (97.90%) Non-Shockable Rhythm: 1338 (1.50%) Shock Delivered: 551 (0.60%) | No: 37926 (97.50%) Non-Shockable Rhythm: 700 (1.80%) Shock Delivered: 291 (0.70%) | <0.001² |
| **Bystander Variables** | **Bystander Witnessed CA** | None: 61530 (47.20%) Non-Medical: 48026 (36.90%) Medical-witnessed: 20702 (15.90%) | None: 45671 (50.00%) Non-Medical: 32308 (35.40%) Medical-witnessed: 13362 (14.60%) | None: 15859 (40.80%) Non-Medical: 15718 (40.40%) Medical-witnessed: 7340 (18.90%) | <0.001² |
| **Bystander Variables** | **Use of AED** | Yes: 106216 (81.50%) No: 24042 (18.50%) | Yes: 74032 (81.10%) No: 17309 (18.90%) | Yes: 32184 (82.70%) No: 6733 (17.30%) | <0.001² |
| **Clinical Interventions** | **Advanced Airway Use** | No: 96824 (74.30%) Yes: 33434 (25.70%) | No: 68368 (74.80%) Yes: 22973 (25.20%) | No: 28456 (73.10%) Yes: 10461 (26.90%) | <0.001² |
| **Clinical Interventions** | **Mechanical Chest Compression** | No: 88114 (67.60%) Yes: 42144 (32.40%) | No: 61474 (67.30%) Yes: 29867 (32.70%) | No: 26640 (68.50%) Yes: 12277 (31.50%) | <0.001² |
| **EMS Timing** | **EMS Total Response Time (minutes)** | 11.6 (7.4-17.5) | 9.8 (6.5-15.6) | 14.9 (11.2-20.1) | <0.001¹ |
| **Location & Time** | **Arrest Location** | Residence (Home): 104842 (80.50%) Public: 13928 (10.70%) Other: 11488 (8.80%) | Residence (Home): 73053 (80.00%) Public: 10105 (11.10%) Other: 8183 (9.00%) | Residence (Home): 31789 (81.70%) Public: 3823 (9.80%) Other: 3305 (8.50%) | <0.001² |
| **Location & Time** | **Daytime of Arrest (hour intervals)** | 8-16: 56834 (43.60%) 16-24: 41274 (31.70%) 0-8: 32150 (24.70%) | 8-16: 40883 (44.80%) 16-24: 28629 (31.30%) 0-8: 21829 (23.90%) | 8-16: 15951 (41.00%) 16-24: 12645 (32.50%) 0-8: 10321 (26.50%) | <0.001² |
| **Location & Time** | **Location (Urban/Rural)** | Urban: 91341 (70.10%) Rural: 38917 (29.90%) | Urban: 91341 (100.00%) | Rural: 38917 (100.00%) | <0.001² |
| **Patient Variables** | **Age** | >65: 83744 (64.30%) ≤65: 46514 (35.70%) | >65: 60372 (66.10%) ≤65: 30969 (33.90%) | >65: 23372 (60.10%) ≤65: 15545 (39.90%) | <0.001² |
| **Patient Variables** | **Initial Cardiac Rhythm** | Asystole: 91127 (70.00%) PEA: 22216 (17.10%) VF/VT: 15648 (12.00%) Bradycardia: 1267 (1.00%) | Asystole: 63367 (69.40%) PEA: 15949 (17.50%) VF/VT: 11138 (12.20%) Bradycardia: 887 (1.00%) | Asystole: 27760 (71.30%) PEA: 6267 (16.10%) VF/VT: 4510 (11.60%) Bradycardia: 380 (1.00%) | <0.001² |
| **Patient Variables** | **OHCA Etiology** | Unknown: 56524 (43.40%) Internal Disease: 35920 (27.60%) Cardiac: 29708 (22.80%) Non-Cardiac: 8106 (6.20%) | Unknown: 41540 (45.50%) Internal Disease: 24474 (26.80%) Cardiac: 19577 (21.40%) Non-Cardiac: 5750 (6.30%) | Unknown: 14984 (38.50%) Internal Disease: 11446 (29.40%) Cardiac: 10131 (26.00%) Non-Cardiac: 2356 (6.10%) | <0.001² |
| **Patient Variables** | **Sex** | Male: 76353 (58.60%) Female: 53905 (41.40%) | Male: 52781 (57.80%) Female: 38560 (42.20%) | Male: 23572 (60.60%) Female: 15345 (39.40%) | <0.001² |

Baseline demographic and clinical characteristics of 130,258 out-of-hospital cardiac arrest cases stratified by urban (n=91,341) versus rural (n=38,917) location. Data are presented as mean±SD, median (IQR), or n (%). P-values calculated by using Mann-Whitney U test for continuous variables (¹) and Chi-square test for categorical variables (²). Abbreviations: IQR, interquartile range; SD, standard deviation.

Supplementary Table 2. Detailed Survival Outcomes by Patient Characteristics

|  | **Variables** | **Total (n=130258)** | **Urban (n=91341)** | **Rural (n=38917)** | **p-value** |
| --- | --- | --- | --- | --- | --- |
| **Overall Survival** |  | 11828 (9.1%, 8.9-9.2) | 8580 (9.4%, 9.2-9.6) | 3248 (8.3%, 8.1-8.6) | <0.001² |
| **By Age** | **<=65** | 5569 (12.0%, 11.7-12.3) | 3916 (12.6%, 12.3-13.0) | 1653 (10.6%, 10.2-11.1) | <0.001² |
|  | **>65** | 6259 (7.5%, 7.3-7.7) | 4664 (7.7%, 7.5-7.9) | 1595 (6.8%, 6.5-7.2) | <0.001² |
| **By Initial Rhythm** | **Asystole** | 4479 (4.9%, 4.8-5.1) | 3210 (5.1%, 4.9-5.2) | 1269 (4.6%, 4.3-4.8) | 0.002² |
|  | **Bradycardia** | 240 (18.9%, 16.9-21.2) | 165 (18.6%, 16.2-21.3) | 75 (19.7%, 16.0-24.0) | 0.693² |
|  | **PEA** | 3301 (14.9%, 14.4-15.3) | 2373 (14.9%, 14.3-15.4) | 928 (14.8%, 13.9-15.7) | 0.910² |
|  | **VF/VT** | 3808 (24.3%, 23.7-25.0) | 2832 (25.4%, 24.6-26.2) | 976 (21.6%, 20.5-22.9) | <0.001² |
| **Bystander Witnessed** | **Medical-witnessed** | 3708 (17.9%, 17.4-18.4) | 2630 (19.7%, 19.0-20.4) | 1078 (14.7%, 13.9-15.5) | <0.001² |
|  | **Non-Medical** | 5501 (11.5%, 11.2-11.7) | 3942 (12.2%, 11.8-12.6) | 1559 (9.9%, 9.5-10.4) | <0.001² |
|  | **None** | 2619 (4.3%, 4.1-4.4) | 2008 (4.4%, 4.2-4.6) | 611 (3.9%, 3.6-4.2) | 0.004² |
| **By Daytime of Arrest (hour intervals)** | **0-8** | 2329 (7.2%, 7.0-7.5) | 1651 (7.6%, 7.2-7.9) | 678 (6.6%, 6.1-7.1) | 0.001² |
|  | **8-16** | 5503 (9.7%, 9.4-9.9) | 4050 (9.9%, 9.6-10.2) | 1453 (9.1%, 8.7-9.6) | 0.004² |
|  | **16-24** | 3996 (9.7%, 9.4-10.0) | 2879 (10.1%, 9.7-10.4) | 1117 (8.8%, 8.4-9.3) | <0.001² |
| **By EMS Response Time** | **≤8 min** | 4541 (12.0%, 11.7-12.3) | 4168 (12.1%, 11.8-12.5) | 373 (10.8%, 9.8-11.9) | 0.029² |
|  | **8-15 min** | 4355 (8.9%, 8.7-9.2) | 2755 (8.5%, 8.2-8.8) | 1600 (9.8%, 9.4-10.3) | <0.001² |
|  | **>15 min** | 2932 (6.7%, 6.5-7.0) | 1657 (6.8%, 6.5-7.1) | 1275 (6.7%, 6.3-7.0) | 0.678² |

Stratified survival rates by key patient characteristics including age, initial cardiac rhythm, bystander witness status, time of day of daytime of Arrest, and EMS response time. Data shows consistent urban-rural disparities across most subgroups.

**Supplementary Table 3. Urban Subgroup Factors Associated with Survival** - Univariable and Multivariable Logistic Regression Results

| **Category** | **Variable** | **Level (Reference)** | **Univariable OR (95% CI)** | **p-value (Uni)** | **Multivariable OR (95% CI)** | **p-value (Multi)** |
| --- | --- | --- | --- | --- | --- | --- |
| **Patient Variables** | **Age** | ≤65 (Ref.) | Ref. | – | – | – |
|  |  | >65 | 0.578 (0.553-0.605) | <0.001 | 0.659 (0.626–0.694) | <0.001 |
|  | **OHCA Etiology** | Cardiac (Ref.) | Ref. | – | – | – |
|  |  | Internal medical Disease | 0.601 (0.570–0.633) | p < 0.001 | 0.784 (0.740–0.832) | <0.001 |
|  |  | Non Cardiac | 0.756 (0.697–0.820) | p < 0.001 | 1.039 (0.950–1.137) | 0.400 |
|  |  | Unknown | 0.118 (0.110–0.127) | p < 0.001 | 0.418 (0.385–0.455) | <0.001 |
|  | **Sex** | Male (Ref.) | Ref. | – | – | – |
|  |  | Female | 0.889 (0.850–0.930) | p < 0.001 | 1.221 (1.159–1.287) | <0.001 |
| **Location & Time** | **Arrest Location** | Public (Ref.) | Ref. | – | – | – |
|  |  | Residence (Home) | 0.405 (0.382–0.429) | p < 0.001 | 0.550 (0.517–0.585) | <0.001 |
|  |  | Other | 0.643 (0.591–0.699) | p < 0.001 | 0.676 (0.616–0.742) | <0.001 |
|  | **Daytime of Arrest (hour intervals)** | 0–8 (Ref.) | Ref. | – | – | – |
|  |  | 8–16 | 1.344 (1.266–1.427) | p < 0.001 | 1.083 (1.019–1.152) | 0.010 |
|  |  | 16–24 | 1.367 (1.283–1.456) | p < 0.001 | 1.109 (1.039–1.184) | 0.002 |
| **Bystander Variables** | **Bystander Witness** | Medical-witnessed (Ref.) | Ref. | – | – | – |
|  |  | Non-Medical | 0.567 (0.537–0.599) | p < 0.001 | 0.549 (0.518–0.582) | <0.001 |
|  |  | None | 0.188 (0.176–0.200) | p < 0.001 | 0.451 (0.420–0.483) | <0.001 |
|  | **Bystander Defibrillation** | No (Ref.) | Ref. | – | – | – |
|  |  | Non-Shockable | 2.266 (1.969–2.608) | p < 0.001 | 1.099 (0.939–1.286) | 0.242 |
|  |  | Shock Delivered | 12.416 (10.481–14.708) | p < 0.001 | 3.489 (2.866–4.248) | <0.001 |
|  | **AED Use** | No (Ref.) | Ref. | – | – | – |
|  |  | Yes | 0.971 (0.917–1.027) | 0.299 | 0.831 (0.785–0.880) | <0.001 |
| **Initial Cardiac Rhythm** | **Initial Rhythm** | Asystole (Ref.) | Ref. | – | – | – |
|  |  | Bradycardia | 4.290 (3.610–5.099) | p < 0.001 | 3.527 (2.896–4.296) | <0.001 |
|  |  | PEA | 3.275 (3.096–3.465) | p < 0.001 | 2.218 (2.087–2.357) | <0.001 |
|  |  | VF/VT | 6.392 (6.047–6.757) | p < 0.001 | 4.648 (4.365–4.949) | <0.001 |
| **EMS Variables** | **Advanced Airway Use** | Advanced Airway (Ref.) | Ref. | – | – | – |
|  |  | No Advanced Airway | 0.089 (0.084–0.093) | p < 0.001 | 0.168 (0.158–0.179) | <0.001 |
|  | **EMS Total Response Time** | ≤8 min (Ref.) | Ref. | – | – | – |
|  |  | 8–15 min | 0.675 (0.642–0.710) | p < 0.001 | 0.755 (0.713–0.798) | <0.001 |
|  |  | >15 min | 0.527 (0.496–0.559) | p < 0.001 | 0.719 (0.673–0.769) | <0.001 |
|  | **Mechanical Chest Compression** | No (Ref.) | Ref. | – | – | – |
|  |  | Yes | 1.691 (1.617–1.769) | p < 0.001 | 0.917 (0.871–0.965) | <0.001 |

Logistic regression analysis results specific to the urban cohort (n=91,341), showing adjusted and unadjusted odds ratios for all variables as predictors of survival. Abbreviations: CI, confidence interval; OR, odds ratio.

**Supplementary Table 4. Rural Subgroup Factors Associated with Survival** - Univariable and Multivariable Logistic Regression Results

| **Category** | **Variable** | **Level (Reference)** | **Univariable OR (95% CI)** | **p-value (Uni)** | **Multivariable OR (95% CI)** | **p-value (Multi)** |
| --- | --- | --- | --- | --- | --- | --- |
| **Patient Variables** | **Age** | ≤65 (Ref.) | Ref. | – | – | – |
|  |  | >65 | 0.616 (0.573-0.662) | <0.001 | 0.627 (0.579–0.679) | <0.001 |
|  | **Etiology** | Cardiac (Ref.) | Ref. | – | – | – |
|  |  | Internal Medical Disease | 0.752 (0.693–0.817) | <0.001 | 0.834 (0.762–0.912) | <0.001 |
|  |  | Non Cardiac | 0.940 (0.823–1.074) | 0.366 | 1.043 (0.902–1.205) | 0.572 |
|  |  | Unknown | 0.148 (0.131–0.167) | <0.001 | 0.427 (0.371–0.492) | <0.001 |
|  | **Sex** | Male (Ref.) | Ref. | – | – | – |
|  |  | Female | 1.064 (0.989–1.145) | 0.097 | 1.280 (1.179–1.389) | <0.001 |
| **Location & Time** | **Arrest Location** | Public (Ref.) | Ref. | – | – | – |
|  |  | Residence (Home) | 0.519 (0.469–0.575) | <0.001 | 0.518 (0.468–0.572) | <0.001 |
|  |  | Other | 0.781 (0.677–0.902) | <0.001 | 0.682 (0.586–0.793) | <0.001 |
|  | **Daytime of Arrest (hour intervals)** | 0–8 (Ref.) | Ref. | – | – | – |
|  |  | 8–16 | 1.426 (1.297–1.567) | <0.001 | 0.998 (0.907–1.098) | 0.972 |
|  |  | 16–24 | 1.379 (1.248–1.522) | <0.001 | 1.008 (0.911–1.115) | 0.884 |
| **Bystander Variables** | **Bystander Witness** | Medical-witnessed (Ref.) | Ref. | – | – | – |
|  |  | Non-Medical | 0.640 (0.589–0.695) | <0.001 | 0.599 (0.548–0.654) | <0.001 |
|  |  | None | 0.233 (0.210–0.258) | <0.001 | 0.502 (0.447–0.563) | <0.001 |
|  | **Bystander Defibrillation** | No (Ref.) | Ref. | – | – | – |
|  |  | Non-Shockable | 2.473 (2.028–3.015) | <0.001 | 1.417 (1.135–1.768) | 0.002 |
|  |  | Shock Delivered | 7.822 (6.170–9.916) | <0.001 | 2.364 (1.796–3.111) | <0.001 |
|  | **AED Use** | No (Ref.) | Ref. | – | – | – |
|  |  | Yes | 1.232 (1.114–1.363) | <0.001 | 0.847 (0.770–0.932) | <0.001 |
| **Initial Cardiac Rhythm** | **Initial Rhythm** | Asystole (Ref.) | Ref. | – | – | – |
|  |  | Bradycardia | 5.135 (3.964–6.651) | <0.001 | 3.756 (2.806–5.028) | <0.001 |
|  |  | PEA | 3.627 (3.316–3.967) | <0.001 | 2.576 (2.341–2.834) | <0.001 |
|  |  | VF/VT | 5.765 (5.266–6.311) | <0.001 | 4.241 (3.839–4.685) | <0.001 |
| **EMS Variables** | **Advanced Airway Use** | Advanced Airway (Ref.) | Ref. | – | – | – |
|  |  | No Advanced Airway | 0.105 (0.097–0.114) | <0.001 | 0.176 (0.160–0.194) | <0.001 |
|  | **EMS Total Response Time** | ≤8 min (Ref.) | Ref. | – | – | – |
|  |  | 8–15 min | 0.896 (0.796–1.010) | 0.072 | 0.698 (0.625–0.780) | <0.001 |
|  |  | >15 min | 0.588 (0.520–0.664) | <0.001 | 0.596 (0.533–0.667) | <0.001 |
|  | **Mechanical Chest Compression** | No (Ref.) | Ref. | – | – | – |
|  |  | Yes | 1.489 (1.383–1.603) | <0.001 | 0.856 (0.789–0.929) | <0.001 |

Logistic regression analysis results specific to the rural cohort (n=38,917), showing adjusted and unadjusted odds ratios for all variables as predictors of survival. Abbreviations: CI, confidence interval; OR, odds ratio.

Supplementary Table 5. Variance Inflation Factors

| **Category** | **Variable** | **Level** | **VIF (Total)** | **VIF (Urban)** | **VIF (Rural)** |
| --- | --- | --- | --- | --- | --- |
| **Patient Variables** | **Age** | >65 | 2.796 | 2.935 | 2.512 |
|  | **Sex** | Female | 1.759 | 1.788 | 1.694 |
|  | **OHCA Etiology** | Internal Disease | 2.121 | 2.159 | 2.055 |
|  |  | Non-Cardiac | 1.241 | 1.261 | 1.203 |
|  |  | Unknown | 3.698 | 3.975 | 3.172 |
| **Initial Cardiac Rhythm** | **Initial Rhythm** | Bradycardia | 1.014 | 1.014 | 1.014 |
|  |  | PEA | 1.250 | 1.258 | 1.232 |
|  |  | VF/VT | 1.189 | 1.193 | 1.179 |
| **EMS Variables** | **Advanced Airway Use** | No Advanced Airway | 5.092 | 5.316 | 4.646 |
|  | **Mechanical Chest Compression** | Yes | 1.496 | 1.506 | 1.477 |
|  | **EMS Total Response Time** | (Continuous) | 1.053 | 1.049 | 1.095 |
| **Location & Time** | **Arrest Location** | Public Area | 1.743 | 1.769 | 1.691 |
|  |  | Residence (Home) | 7.067 | 7.065 | 7.142 |
| **Bystander Variables** | **Bystander Witness** | Non-Medical | 2.985 | 3.042 | 2.883 |
|  |  | None | 4.167 | 4.560 | 3.412 |
|  | **Bystander Defibrillation** | Non-Shockable | 1.052 | 1.052 | 1.054 |
|  |  | Shock Delivered | 1.027 | 1.027 | 1.028 |
|  | **AED Use** | Yes | 4.591 | 4.513 | 4.820 |

Assessment of multicollinearity in the regression model by using variance inflation factors (VIF). A VIF < 10 indicates acceptable multicollinearity. The highest VIF was observed for residential location (7.07), followed by advanced airway management (5.09). Abbreviations: VIF, variance inflation factor.

**Supplementary Table 6. Covariate Balance Before and After Propensity Score Matching**

| **Variable** | **Level** | **Pre- Urban** | **Pre-Rural** | **Pre SMD** | **Pre p-value** | **Post Urban** | **Post Rural** | **Post SMD** | **Post p-value** |
| --- | --- | --- | --- | --- | --- | --- | --- | --- | --- |
| **Bystander Witnessed** | **Medical-witnessed** | 13,362 (14.6%) | 7,340 (18.9%) | -0.11 | <0.001 | 8,623 (22.3%) | 7,318 (18.9%) | 0.08 | <0.001 |
|  | **Non-Medical** | 32,308 (35.4%) | 15,718 (40.4%) | -0.10 | <0.001 | 16,706 (43.1%) | 15,557 (40.2%) | 0.06 | <0.001 |
|  | **No Data/Unknown** | 45,671 (50.0%) | 15,859 (40.8%) | 0.19 | <0.001 | 13,405 (34.6%) | 15,859 (40.9%) | -0.13 | <0.001 |
| **OHCA Etiology** | **Cardiac** | 19,577 (21.4%) | 10,131 (26.0%) | -0.11 | <0.001 | 11,386 (29.4%) | 9,970 (25.7%) | 0.08 | <0.001 |
|  | **Internal Disease** | 24,474 (26.8%) | 11,446 (29.4%) | -0.06 | <0.001 | 11,838 (30.6%) | 11,424 (29.5%) | 0.02 | 0.001 |
|  | **Non-Cardiac** | 5,750 (6.3%) | 2,356 (6.1%) | 0.01 | 0.102 | 2,342 (6.0%) | 2,356 (6.1%) | -0.00 | 0.845 |
|  | **Unknown** | 41,540 (45.5%) | 14,984 (38.5%) | 0.14 | <0.001 | 13,168 (34.0%) | 14,984 (38.7%) | -0.10 | <0.001 |
| **Age** | **>65** | 60,372 (66.1%) | 23,372 (60.1%) | 0.13 | <0.001 | 21,634 (55.9%) | 23,316 (60.2%) | -0.09 | <0.001 |
| **Initial Cardiac Rhythm** | **Bradycardia** | 887 (1.0%) | 380 (1.0%) | -0.00 | 0.953 | 375 (1.0%) | 380 (1.0%) | -0.00 | 0.884 |
|  | **PEA** | 15,949 (17.5%) | 6,267 (16.1%) | 0.04 | <0.001 | 6,095 (15.7%) | 6,259 (16.2%) | -0.01 | 0.110 |
|  | **VF/VT** | 11,138 (12.2%) | 4,510 (11.6%) | 0.02 | 0.002 | 4,662 (12.0%) | 4,504 (11.6%) | 0.01 | 0.081 |
| **Daytime of Arrest (hour intervals)** | **16–24** | 28,629 (31.3%) | 12,645 (32.5%) | -0.02 | <0.001 | 12,882 (33.3%) | 12,571 (32.5%) | 0.02 | 0.018 |
|  | **8–16** | 40,883 (44.8%) | 15,951 (41.0%) | 0.08 | <0.001 | 15,645 (40.4%) | 15,909 (41.1%) | -0.01 | 0.054 |
| **Sex** | **Female** | 38,560 (42.2%) | 15,345 (39.4%) | 0.06 | <0.001 | 14,244 (36.8%) | 15,331 (39.6%) | -0.06 | <0.001 |
| **Arrest Location** | **Public Area** | 10,105 (11.1%) | 3,823 (9.8%) | 0.04 | <0.001 | 3,627 (9.4%) | 3,823 (9.9%) | -0.02 | 0.017 |
|  | **Residence (Home)** | 73,053 (80.0%) | 31,789 (81.7%) | -0.04 | <0.001 | 31,936 (82.4%) | 31,608 (81.6%) | 0.02 | 0.002 |
| **Bystander Defibrillation** | **Non-Shockable Rhythm** | 1,338 (1.5%) | 700 (1.8%) | -0.03 | <0.001 | 796 (2.1%) | 695 (1.8%) | 0.02 | 0.009 |
|  | **Shock Delivered** | 551 (0.6%) | 291 (0.7%) | -0.02 | 0.003 | 347 (0.9%) | 290 (0.7%) | 0.02 | 0.026 |
| **Use of AED** | **Yes** | 74,032 (81.1%) | 32,184 (82.7%) | -0.04 | <0.001 | 32,504 (83.9%) | 32,002 (82.6%) | 0.03 | <0.001 |
| **Advanced Airway** | **No Advanced Airway** | 68,368 (74.8%) | 28,456 (73.1%) | 0.04 | <0.001 | 27,532 (71.1%) | 28,294 (73.0%) | -0.04 | <0.001 |
| **Mechanical Chest Compression** | **Yes** | 29,867 (32.7%) | 12,277 (31.5%) | 0.02 | <0.001 | 12,170 (31.4%) | 12,261 (31.7%) | -0.01 | 0.486 |
| **EMS Total Response Time** | **Time (mean ± SD)** | -0.1 ± 1.0 | 0.2 ± 1.0 | -0.29 | <0.001 | 0.3 ± 1.0 | 0.2 ± 1.0 | 0.07 | <0.001 |
|  | **≤ 8 min** | 34,381 (37.6%) | 3,428 (8.8%) | 0.73 | <0.001 | 2,860 (7.4%) | 3,428 (8.9%) | -0.05 | <0.001 |
|  | **8–15 min** | 32,391 (35.5%) | 16,311 (41.9%) | -0.13 | <0.001 | 14,498 (37.4%) | 16,311 (42.1%) | -0.10 | <0.001 |
|  | **> 15 min** | 24,513 (26.8%) | 19,159 (49.2%) | -0.47 | <0.001 | 21,351 (55.1%) | 18,976 (49.0%) | 0.12 | <0.001 |

Standardized mean differences (SMD) for key baseline characteristics before and after propensity score matching. A standardized difference <0.1 indicates adequate balance. Abbreviations: Std..diff, standardized difference.

Supplementary Table 7. Cross-Validation Results

| **Cross-Validation** | **Metric** | **Mean** | **Std** | **CI Lower** | **CI Upper** |
| --- | --- | --- | --- | --- | --- |
|  | AUC | 0.764 | 0.007 | 0.751 | 0.777 |
|  | Brier Score | 0.130 | 0.001 | 0.128 | 0.132 |
| **Bootstrap Validation** | **Metric** | **Apparent** | **Optimism** | **CI Lower** | **CI Upper** |
|  | AUC | 0.764 | 0.00009 | 0.760 | 0.769 |
|  | Brier Score | 0.130 | 0.000001 | 0.129 | 0.131 |

Performance metrics from internal validation through Cross-Validation (Internal Validation) and Bootstrap validation (External Validation). AUC = area under the curve; CI = confidence interval.

Supplementary Table 8. Propensity Score Model Coefficients

| **Category** | **Variable** | **Level** | **Coefficient** | **Importance** |
| --- | --- | --- | --- | --- |
| **Patient Variables** | **Age** | >65 | 0.258 | 0.258 |
|  | **Sex** | Female | 0.089 | 0.089 |
|  | **OHCA Etiology** | Cardiac | -0.191 | 0.191 |
|  |  | Internal Disease | -0.006 | 0.006 |
|  |  | Non-Cardiac | 0.108 | 0.108 |
|  |  | Unknown | 0.280 | 0.280 |
| **Location & Time** | **Arrest Location** | Public | 0.031 | 0.031 |
|  |  | Residence (Home) | -0.126 | 0.126 |
| **Bystander Variables** | **Bystander Witness** | Medical-witnessed | -0.132 | 0.132 |
|  |  | Non-Medical | -0.015 | 0.015 |
|  |  | None | 0.338 | 0.338 |
| **Initial Cardiac Rhythm** | **Initial Rhythm** | Bradycardia | 0.043 | 0.043 |
|  |  | PEA | 0.071 | 0.071 |
|  |  | VF/VT | 0.039 | 0.039 |
| **Bystander Variables** | **Bystander Defibrillation** | Non-Shockable | 0.024 | 0.024 |
|  |  | Shock Delivered | -0.043 | 0.043 |
|  | **AED Use** | Yes | -0.132 | 0.132 |
| **EMS Variables** | **Advanced Airway Use** | No Advanced Airway | -0.096 | 0.096 |
|  | **Mechanical Chest Compression** | Yes | 0.062 | 0.062 |
|  | **Daytime of Arrest (hour intervals)** | 16-24 | -0.022 | 0.022 |
|  |  | 8-16 | 0.011 | 0.011 |
|  | **EMS Total Response Time** | (Continuous) | 0.052 | 0.052 |
|  | **Response Time Category** | ≤8 min | 1.171 | 1.171 |
|  |  | 8-15 min | -0.495 | 0.495 |
|  |  | >15 min | -1.087 | 1.087 |

Coefficients and relative importance of variables in the propensity score model are used for matching urban and rural cases. Higher absolute coefficient values indicate a stronger influence on the propensity score. The importance values represent the normalized contribution of each variable to the model's predictive power for urban/rural location, and larger values indicate greater importance in distinguishing between locations. Reference categories are omitted from the table and have coefficient values of 0. Continuous variables such as EMS Total Response Time are modeled both as continuous values and as categorized thresholds to capture non-linear effects.
